# Efficacy and safety of recombinant von Willebrand factor in on-demand treatment of children with von Willebrand disease: up to 4 years of phase 3/3b follow-up

**DOI:** 10.1016/j.rpth.2026.106817

**Published:** 2026-07-13

**Authors:** Shayla Bergmann, Sanjay Ahuja, Canan Albayrak, Marjon H. Cnossen, Amy L. Dunn, Veerle Labarque, Matteo Luciani, Christoph Male, Eric S. Mullins, Sophie Susen, Pascual Marco Vera, Ali G. Mokdad, Yi Wang, Josh Weng, Jingmei Zhang

**Affiliations:** 1 Department of Pediatrics, Medical University of South Carolina, Charleston, South Carolina, USA; 2 Innovative Hematology Inc., Indiana Hemophilia and Thrombosis Center, Indianapolis, Indiana, USA; 3 Division of Pediatric Hematology and Oncology, Ondokuz Mayis University Faculty of Medicine, Samsun, Turkey; 4 Department of Pediatric Hematology and Oncology, Erasmus Medical Center Sophia Children’s Hospital, University Medical Center Rotterdam, Rotterdam, the Netherlands; 5 Department of Hematology, Oncology, Bone Marrow Transplant, Nationwide Children’s Hospital and The Ohio State University College of Medicine, Columbus, Ohio, USA; 6 Department of Pediatric Hemato-Oncology, University Hospitals Leuven, Centre of Molecular and Vascular Biology, Katholieke Universiteit Leuven, Leuven, Belgium; 7 Onco-Hematology, Cell and Gene Therapy and Bone Marrow Transplant Clinic Area, Bambino Gesù Children’s Hospital, Istituto di Ricovero e Cura a Carattere Scientifico, Rome, Italy; 8 Department of Paediatrics, Medical University of Vienna, Vienna, Austria; 9 Division of Hematology, Cincinnati Children’s Hospital Medical Center, University of Cincinnati College of Medicine, Cincinnati, Ohio, USA; 10 University of Lille, Inserm, Centre Hospitalier Universitaire Lille, Institut Pasteur de Lille, Lille, France; 11 Biomedical Research Institute (Instituto de Investigación Sanitaria y Biomédica de Alicante), Clinical Medicine Department, Miguel Hernández University, Alicante, Spain; 12 Takeda Development Center Americas Inc, Cambridge, Massachusetts, USA

**Keywords:** clinical trial, factor VIII, pediatrics, von Willebrand disease, von Willebrand factor

## Abstract

**Background:**

Recombinant von Willebrand factor (rVWF) is approved to treat von Willebrand disease (VWD) in adults and was recently approved in the United States for pediatric on-demand and perioperative treatment.

**Objectives:**

To evaluate the efficacy and safety of rVWF, without or with recombinant factor VIII (rFVIII), for on-demand treatment of bleeding events (BEs) in pediatric patients with VWD.

**Methods:**

A prospective, open-label, phase 3 study (NCT02932618) evaluated efficacy and safety of on-demand rVWF, without or with rFVIII, for treating nonsurgical BEs (for up to 18 months) in patients aged <18 years with severe VWD (VWF:ristocetin cofactor <20 IU/dL). The primary end point was treatment success (mean efficacy, <2.5; 4-point scale, 1 [excellent] to 4 [none]). Patients completing the study could enter a phase 3b continuation study (NCT03879135), evaluating safety and efficacy of rVWF for 3 additional years.

**Results:**

Of 25 patients in the phase 3 study, 11 (44%) had type 3 VWD. Eighteen patients reported 104 BEs treated with on-demand rVWF; all were treated successfully (mean [SD] efficacy, 1.01 [0.04]). All 98 BEs with efficacy ratings were rated excellent or good. Most BEs (82%) were treated with 1 rVWF infusion; 27% were treated with additional rFVIII. rVWF was well tolerated, with no adverse events leading to study or treatment discontinuation. No thromboembolic events, severe hypersensitivity reactions, or treatment-related serious adverse events occurred. No neutralizing or binding antibodies to rVWF were observed. Efficacy and tolerability of on-demand rVWF were maintained in the continuation study.

**Conclusion:**

rVWF, without or with rFVIII, was efficacious for treating nonsurgical BEs in pediatric patients with severe VWD, with efficacy maintained for up to 4 years and no new safety findings.

## Essentials

• VWD in children can cause serious bleeding and impact quality of life.
• This study tested rVWF for bleeds in children with VWD.
• All treated bleeds were controlled, mostly with 1 dose; results were maintained up to 4 years.
• rVWF was well tolerated, and side effects were similar to those seen in other studies of rVWF.

## Graphical abstract

**Figure:**
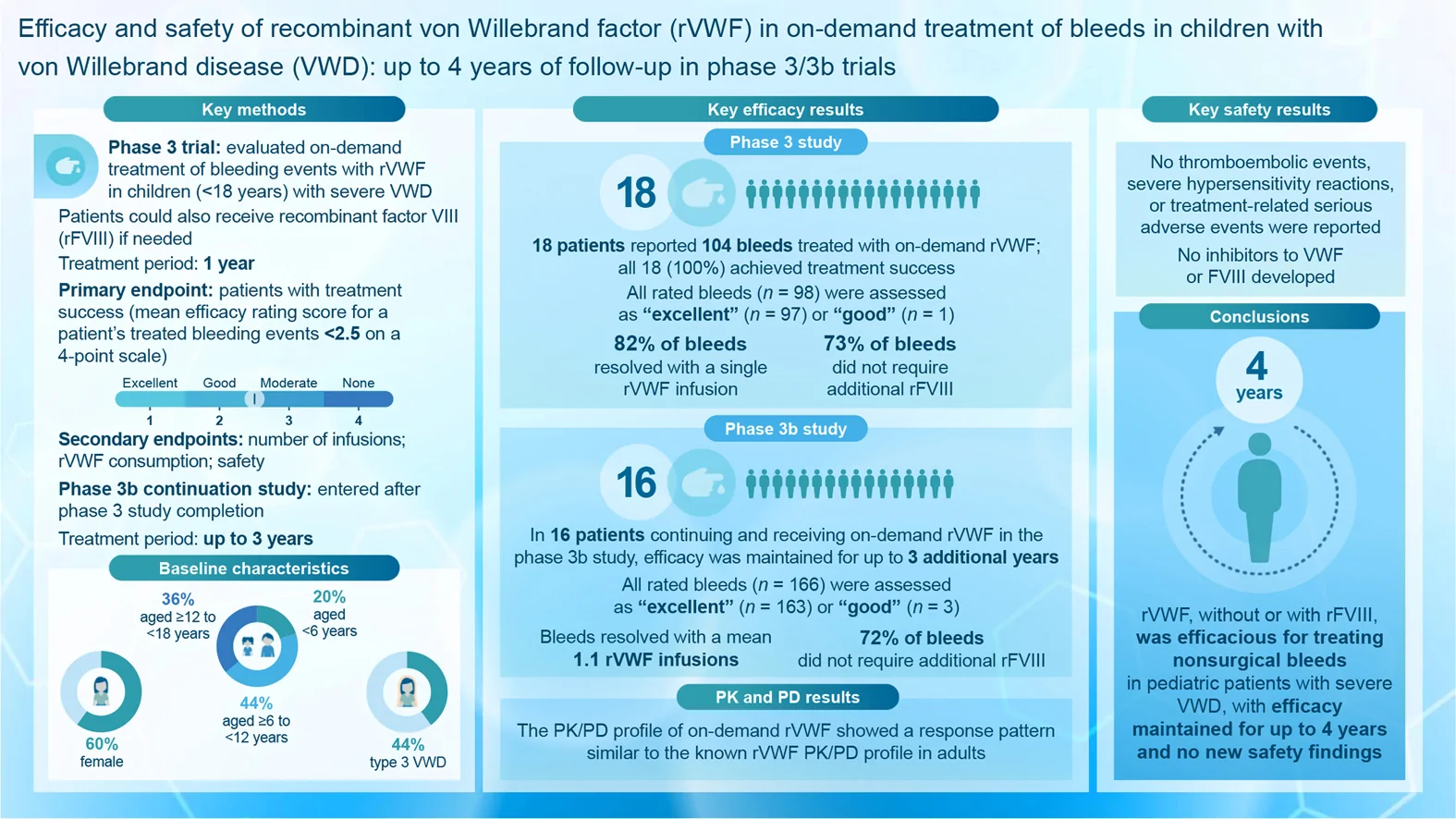

## 1 Introduction

von Willebrand disease (VWD) has an estimated incidence ranging between 109 and 1300 per 100,000 in pediatric populations [[1], [2], [3]]. It is caused by quantitative and/or qualitative defects of von Willebrand factor (VWF), which mediates platelet adhesion and stabilizes coagulation factor (F)VIII [4,5]. The disease is classified into 3 types: type 1 (partial deficiency of VWF); type 2 (qualitative defects of VWF classified into 2A, 2B, 2M, and 2N subtypes); and type 3 (complete or almost complete absence of VWF) [4].

Diagnosing VWD in children can be challenging because younger patients may not yet have encountered major hemostatic challenges such as surgery or dental extraction, and mild cases often show borderline findings [6,7]. In addition, bleeding symptoms are frequently nonspecific and may overlap with normal childhood bruising or nosebleeds [7]. Diagnosis is further complicated by individual fluctuations in VWF levels related to age, stress, exercise, and illness [4]. Therefore, delayed diagnosis or misdiagnosis of VWD is common, even in symptomatic patients, prolonging the time before children with the condition receive appropriate treatment [8,9]. However, symptoms due to VWD in childhood, from severe to nuisance bleeding, may place a considerable burden on parents and caregivers [10,11]. VWD negatively affects health-related quality of life in children, impacting physical, behavioral, and emotional functioning compared with those without VWD [12]. First bleeds in children with VWD commonly include epistaxis, oropharyngeal bleeding, or cutaneous bleeding [7,13]. Furthermore, joint bleeds in patients with moderate and severe VWD have been reported to mostly start before 16 years of age [14].

Patients with VWD, particularly those with a more severe bleeding phenotype, have an increased risk of bleeding; this includes serious and potentially life-threatening bleeding events (BEs) and milder BEs requiring on-demand medication [15,16]. Treatment options for pediatric patients with VWD include hormonal therapy in adolescent girls, antifibrinolytics, desmopressin (DDAVP; when DDAVP response is expected and sufficient and the child is aged ≥2 to 4 years [17]), and replacement therapy with VWF concentrates [18]. A recent real-world study of 117 pediatric patients with VWD in a French registry reported that 50% of the children received antifibrinolytics, 11% received DDAVP, 26% of girls received hormonal therapy, and 4% of all patients received long-term prophylaxis with VWF [18].

Recombinant VWF (rVWF; vonicog alfa; VEYVONDI/VONVENDI) is approved for the management of VWD in adults [19,20]; rVWF was also recently approved for on-demand treatment (in the United States, European Union, United Kingdom, and Japan [[19], [20], [21], [22]]) and perioperative management (in the United States and Japan [20,21]) of pediatric patients with VWD. The properties and characteristics of rVWF make it a valuable treatment option for patients with VWD, including children. rVWF is produced using recombinant DNA technology, eliminating the risk of blood-borne pathogen transmission [23]. Compared with plasma-derived VWF products, rVWF also has a longer half-life and exhibits higher specific activity [[24], [25], [26]]. Unlike plasma-derived VWF, rVWF contains the full spectrum of multimers, including ultralarge multimers with higher biological activity [23,27]. In addition, rVWF does not contain FVIII, allowing more flexibility of dosing and reducing concerns relating to excessive FVIII levels in specific patient groups [23,28,29].

To assess the efficacy, safety, pharmacokinetics (PK), and pharmacodynamics (PD) of rVWF as an on-demand therapy to control BEs, and for elective and emergency surgery coverage, in pediatric patients with severe VWD, a prospective, open-label, phase 3 study is being conducted at 45 sites in 13 countries in Europe and the United States (ClinicalTrials.gov, NCT02932618). In this study, we present final data from the completed on-demand treatment arm following a planned interim analysis of the phase 3 study (results from the surgery arms will be reported separately once completed). Data are also presented from pediatric patients who completed the phase 3 study and continued on-demand treatment in a phase 3b continuation study (ClinicalTrials.gov, NCT03879135).

## 2 Methods

### 2.1 Study design

The pivotal study (ClinicalTrials.gov, NCT02932618; registered October 13, 2016) was a phase 3, prospective, multicenter, uncontrolled, open-label clinical trial (Supplementary Figure 1). Patients in the on-demand treatment arm received rVWF treatment for nonsurgical BEs for 12 months (up to 18 months for some patients who were waiting to enter the continuation study [NCT03879135]).

Patients were recruited into 3 age cohorts: ≥12 to <18 years, ≥6 to <12 years, and <6 years. Enrollment started in the oldest age group and was opened to patients aged ≥6 to <12 years after the data monitoring committee reviewed the PK/PD results from 6 patients in the first age group. Enrollment of patients aged <6 years was opened after the committee reviewed PK results from 6 patients in the ≥6- to <12-year age group. Enrollment of previously untreated patients could not start until 4 previously treated patients were treated with rVWF. A planned interim analysis was conducted when the on-demand treatment arm was complete and is presented in this study.

At baseline visit, PK/PD of rVWF (VWF ristocetin cofactor [VWF:RCo], VWF antigen [VWF:Ag], VWF collagen-binding capacity [VWF:CB], and FVIII coagulation activity [FVIII:C]) were measured following an infusion of 50 ± 5 IU/kg rVWF. For on-demand treatment of nonsurgical BEs, an initial dose of 40 to 60 IU/kg rVWF (up to 80 IU/kg for major BEs) was infused, along with 30 to 45 IU/kg recombinant FVIII (rFVIII; octocog alfa; ADVATE) for patients without hemostatically effective baseline FVIII levels, at the investigator’s discretion. Subsequent rVWF doses were administered every 8 to 24 hours, without or with additional rFVIII, to maintain VWF:RCo and FVIII levels for as long as deemed necessary by the investigator; rFVIII was only to be administered if plasma FVIII levels fell <30 IU/dL during the treatment period.

The study was conducted in accordance with the Declaration of Helsinki and applicable national and local regulatory requirements. The study protocol, protocol amendments, informed consent form, and any other written information were reviewed and approved/given favorable opinion by the ethics committee and applicable regulatory authorities before implementation.

### 2.2 Patient population

Eligible patients were children and adolescents (aged 0 to <18 years) with severe VWD (VWF:RCo <20 IU/dL) who were unable to tolerate or were inadequately responsive to DDAVP. Patients provided assent (if appropriate), and their legally authorized representative(s) provided informed consent. If applicable, patients agreed to use adequate birth control measures and female patients of childbearing potential were required to have a negative pregnancy test before enrollment. Key exclusion criteria included diagnosis of pseudo-VWD or other hereditary or acquired coagulation disorders; a history or presence of a VWF inhibitor or a FVIII inhibitor; known hypersensitivity to any of the components of the study drug; and a medical history of a thromboembolic event. Patients who were pregnant or lactating at the time of informed consent or assent were also excluded. A full list of inclusion and exclusion criteria is provided in Supplementary Table 1.

### 2.3 Study end points

The primary efficacy end point was the number and percentage of pediatric patients with treatment success for rVWF-treated nonsurgical BEs, defined as a mean efficacy rating score of <2.5 on a 4-point scale (1, excellent; 2, good; 3, moderate; and 4, none) (Supplementary Table 2). Severity of BEs was assessed by the investigators and determined based on clinical expertise and judgment. Secondary efficacy end points included the number of treated nonsurgical BEs with an efficacy rating of excellent or good, and the number of infusions, rVWF units, and rFVIII units (if needed) per BE. Safety was assessed in terms of the incidence and severity of treatment-emergent adverse events (TEAEs), the incidence of thrombotic events and severe hypersensitivity reactions, and immunogenicity, which included the development of neutralizing antibodies (inhibitors) to VWF and FVIII, total binding antibodies to VWF, and binding antibodies to Chinese hamster ovary proteins, murine immunoglobulin (Ig)G, and recombinant furin.

### 2.4 Analyses

Final data are presented for the on-demand treatment arm of the phase 3 study (November 6, 2017, to data cutoff December 1, 2023). A target sample size of ≥24 pediatric patients with severe VWD (8 in each age cohort) was based on the Guideline on the Clinical Investigation of Human Plasma Derived von Willebrand Factor Products [30] and was not based on a power calculation for a significance test.

The full analysis set (FAS) included all patients who were enrolled and met all eligibility criteria, had received any amount of study drug, and had provided ≥1 hemostatic assessment within 24 hours of an rVWF infusion, without or with rFVIII. All primary and secondary efficacy end points were assessed in the FAS. The safety analysis set (SAS) was used for the analyses of all safety end points and included all enrolled patients who received ≥1 dose of rVWF, without or with rFVIII.

PK/PD properties were assessed in the PK analysis set, defined as all enrolled patients who were not actively bleeding at the time of the PK infusion, had no BEs during the PK assessment, and had ≥1 quantifiable postdose PK measurement. Patients had an initial PK assessment at the baseline visit after a washout of ≥168 hours before infusion of 50 ± 5 IU/kg rVWF. PD was assessed by evaluating FVIII levels, as a marker of treatment effect. Sparse PK/PD samples were collected preinfusion and over 96 hours at 3 time points after the PK infusion, with patients in the 3 age cohorts randomized separately into 1 of 3 different sampling sequences to ensure that the samples were equally distributed among patients. Median concentration values at each scheduled time point were calculated. Point estimates by age cohort and overall were presented for area under the plasma concentration/time curve from 0 to 96 hours post infusion (AUC_0-96h_), maximal plasma concentration (*C*_max_), and time to maximal plasma concentration (*T*_max_) PK/PD parameters, with and without preinfusion correction. These parameters were derived using the noncompartmental estimation approach for sparse sampling designs.

Descriptive statistics are presented for demographic and baseline characteristics, and primary, secondary, and exploratory end points. Data are presented as observed, with no imputation for missing data. For the primary efficacy end point, point estimates and corresponding 2-sided exact 95% CIs for the percentage of patients with treatment success were calculated using the Clopper–Pearson method. The analysis for the primary outcome measure did not include inferential testing of statistical hypotheses.

### 2.5 Rollover pediatric on-demand treatment cohort in the continuation study

Patients in the on-demand arm who completed the phase 3 study had the option to enter a continuation study (ClinicalTrials.gov, NCT03879135; registered March 18, 2019) (Supplementary Figure 1) to extend the on-demand treatment with the study drug for up to an additional 3 years. This was a phase 3b, prospective, open-label, uncontrolled, multicenter study evaluating the long-term safety and efficacy of rVWF in pediatric and adult patients with severe VWD. This analysis includes all patients who completed the phase 3 study and chose to rollover for extended on-demand treatment (rollover pediatric on-demand treatment cohort) in the phase 3b study. The screening visit for the continuation study coincided with the completion visit of the phase 3 study upon receipt of informed consent. Patients were followed up for a maximum of 3 years (minimum 1 year) or until rVWF became commercially available, whichever occurred first.

Efficacy end points included hemostatic efficacy rating for the on-demand treatment of BEs and study drug consumption. Safety was assessed by the incidence and severity of TEAEs and the incidences of thrombotic events, severe hypersensitivity reactions, and immunogenicity. Safety data were evaluated in the SAS, defined as all patients who received rVWF. Efficacy analyses were conducted on the FAS, defined as all patients in the SAS who met all eligibility criteria. Data are presented descriptively; no formal statistical tests were carried out for this study.

## 3 Results

### 3.1 Patient disposition and demographics

**Figure 1:**
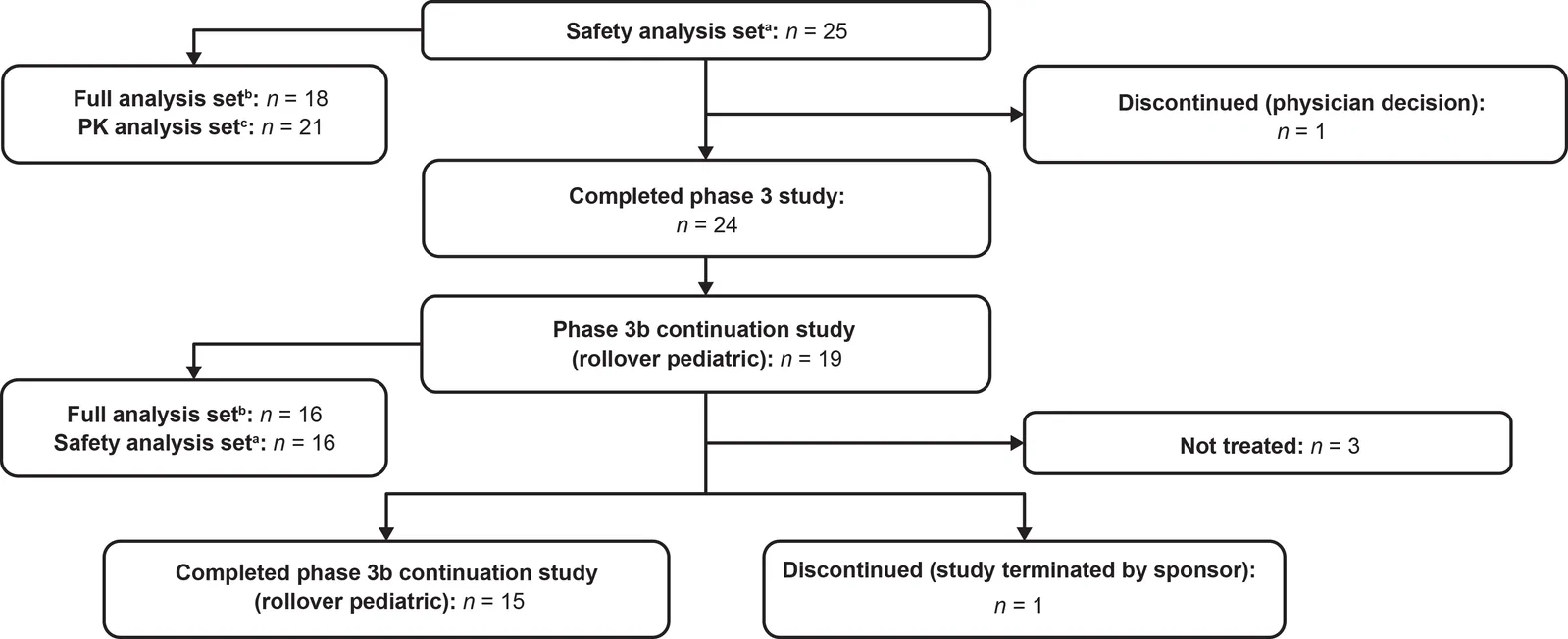
Patient disposition. ^a^All enrolled patients who received any infusion of recombinant von Willebrand factor. ^b^All enrolled patients met all eligibility criteria, had received any amount of study drug, and provided ≥1 hemostatic assessment within 24 hours of a study drug infusion. ^c^All patients who completed the required washout, received an on-demand infusion followed by PK analysis, were not actively bleeding at the time of the recombinant von Willebrand factor infusion, were not bleeding during the PK assessment, and had ≥1 quantifiable postdose measurement. PK, pharmacokinetic.

In the phase 3 study, 25 eligible patients (female, *n* = 15 [60%]; male, *n* = 10 [40%]) were enrolled in the on-demand treatment arm, received ≥1 dose of rVWF, and were included in the SAS (Figure 1) (≥12 to <18 years, *n* = 9 [36%]; ≥6 to <12 years, *n* = 11 [44%]; <6 years, *n* = 5 [20%]). The numbers of patients with VWD types 1, 2, and 3 were 5 (20%), 9 (36%), and 11 (44%), respectively. Eighteen of the 25 patients who experienced ≥1 bleeding event that was treated with rVWF were included in the FAS. Baseline characteristics are shown in Table 1 for the SAS and in Supplementary Table 3 for the FAS. Distribution of VWD types by age cohort in the SAS is shown in Table 2. Twenty-four patients completed the study after a treatment period of ∼12 to 17 months. One patient discontinued the study at 10.5 months because of the physician’s decision to start prophylaxis.

**Table 1:** Baseline characteristics (safety analysis set).

Characteristic	Phase 3 study (*N* = 25)	Phase 3b rollover pediatric OD treatment cohort (*N* = 16)
Age (y)		
Mean (SD)	10.2 (5.2)	10.4 (5.4)
Median (range)	11.0 (1-17)	10.5 (2-18)
Age category		
18 y	0	2 (13)
≥12 to <18 y	9 (36)	6 (38)
≥6 to <12 y	11 (44)	5 (31)
<6 y	5 (20)	3 (19)
Sex		
Female	15 (60)	9 (56)
Male	10 (40)	7 (44)
Female patient of childbearing potential^a^		
Yes	6 (40)	3 (33)
VWD type		
1	5 (20)	4 (25)
2A	6 (24)	1 (6)
2B	3 (12)	3 (19)
3	11 (44)	8 (50)
Body mass index (kg/m^2^)		
Mean (SD)	20.2 (6.3)	21.7 (7.8)
Median (range)	18.6 (12.8-40.7)	19.9 (13.4-40.6)
Race^b^		
White	21 (91)	14 (93)
Asian	1 (4)	0
Multiple	1 (4)	1 (7)
Not reported	2	1
Ethnicity^b^		
Hispanic or Latino	3 (13)	1 (7)
Not Hispanic or Latino	21 (88)	14 (93)
Not reported	1	1

Values are *n* (%) unless specified.

OD, on-demand; VWD, von Willebrand disease.

a Percentages based on number of female patients.

b Percentages calculated with the missing data numbers removed.

**Table 2:** VWD type by age group (safety analysis set).

VWD type, *n* (%)	Phase 3 study (*N* = 25)			Phase 3b rollover pediatric OD treatment cohort (*N* = 16)^a^		
	Age ≥12 to <18 y (*n* = 9)	Age ≥6 to <12 y (*n* = 11)	Age <6 y (*n* = 5)	Age ≥12 to ≤18 y (*n* = 8)	Age ≥6 to <12 y (*n* = 5)	Age <6 y (*n* = 3)
1	0	2 (18)	3 (60)	1 (13)	0	3 (100)
2A	4 (44)	2 (18)	0	1 (13)	0	0
2B	1 (11)	2 (18)	0	2 (25)	1 (20)	0
3	4 (44)	5 (45)	2 (40)	4 (50)	4 (80)	0

OD, on-demand; VWD, von Willebrand disease.

a The differing age distributions of VWD subtypes between the phase 3 study and the phase 3b rollover pediatric OD treatment cohort are due to patients aging into the next category during the study period, as well as some patients not transitioning to the phase 3b study rollover OD treatment cohort. For the patients who did not roll over, this was due to the lack of a need for treatment (ie, no bleeding events required treatment in the phase 3 study) and concern about study burden.

Of the 24 patients who completed the on-demand treatment arm of the phase 3 study, 19 transitioned into the phase 3b study and continued for up to 3 additional years in the rollover on-demand treatment cohort (see further for phase 3b study results).

### 3.2 Hemostatic efficacy

Overall, in the phase 3 study, 104 nonsurgical BEs (minor/mild, *n* = 48; moderate, *n* = 31; major/severe, *n* = 2; unknown severity, *n* = 23) were treated with rVWF (without or with rFVIII) in 18 patients in the on-demand treatment arm. BEs were categorized as traumatic (52 BEs [50%]), spontaneous (33 BEs [32%]), menstrual (11 BEs [11%]), and unknown (8 BEs [8%]). Of 104 treated bleeds, 57 (55%) were reported in patients aged ≥12 to <18 years, 37 (36%) in patients aged ≥6 to <12 years, and 10 (10%) in patients aged <6 years. Fifty (48%) of the total 104 bleeds were reported in patients with type 3 VWD (Table 3). Most BEs were mucosal (nasopharyngeal, mouth/oral cavity, and menstrual/menorrhagia; 51 BEs; 49%) or joint bleeds (17 BEs; 16%) (Supplementary Table 4).

**Table 3:** Hemostatic efficacy of on-demand treatment in the phase 3 study (full analysis set).

Parameter	Full analysis set (*N* = 18)	Age group (y)			VWD type			
		≥12 to <18 (*n* = 6)	≥6 to <12 (*n* = 9)	<6 (*n* = 3)	Type 1 (*n* = 2)	Type 2A (*n* = 3)	Type 2B (*n* = 2)	Type 3 (*n* = 11)
Treated nonsurgical bleeding events (*n*)	104	57	37	10	12	37	5	50
Bleeding events per patient								
Mean (SD)	5.8 (5.0)	9.5 (6.9)	4.1 (2.7)	3.3 (0.6)	6.0 (4.2)	12.3 (9.5)	2.5 (0.7)	4.5 (2.3)
Median (Q1, Q3)	4.0 (3.0, 7.0)	7.5 (5.0, 12.0)	3.0 (2.0, 6.0)	3.0 (3.0, 4.0)	6.0 (3.0, 9.0)	12.0 (3.0, 22.0)	2.5 (2.0, 3.0)	4.0 (3.0, 6.0)
Treatment success^a^								
*n* (%)	18 (100)	6 (100)	9 (100)	3 (100)	2 (100)	3 (100)	2 (100)	11 (100)
95% CI	81.5-100	54.1-100	66.4-100	29.2-100	15.8-100	29.2-100	15.8-100	71.5-100
Efficacy rating score per patient								
Mean (SD)	1.01 (0.04)	1.00 (0.00)	1.02 (0.06)	1.00 (0.00)	1.00 (0.00)	1.00 (0.00)	1.00 (0.00)	1.02 (0.05)
Median (Q1, Q3)	1.0 (1.0, 1.0)	1.0 (1.0, 1.0)	1.0 (1.0, 1.0)	1.0 (1.0, 1.0)	1.0 (1.0, 1.0)	1.0 (1.0, 1.0)	1.0 (1.0, 1.0)	1.0 (1.0, 1.0)
Bleeding events with efficacy rating of good/excellent								
Bleeding events with a known efficacy rating (*n*)	98	52	36	10	12	32	5	49
*n* (%)^b^	98 (100)	52 (100)	36 (100)	10 (100)	12 (100)	32 (100)	5 (100)	49 (100)
95% CI	96.3-100	93.2-100	90.3-100	69.2-100	73.5-100	89.1-100	47.8-100	92.7-100

Q, quartile; VWD, von Willebrand disease.

a Primary end point defined as a mean efficacy rating score of <2.5 for bleeding events treated with recombinant von Willebrand factor using a 4-point scale (1, excellent; 2, good; 3, moderate; 4, none).

b Investigator-assessed hemostatic efficacy of excellent/good at resolution of bleed by location is shown in Supplementary Table 4.

**Figure 2:**
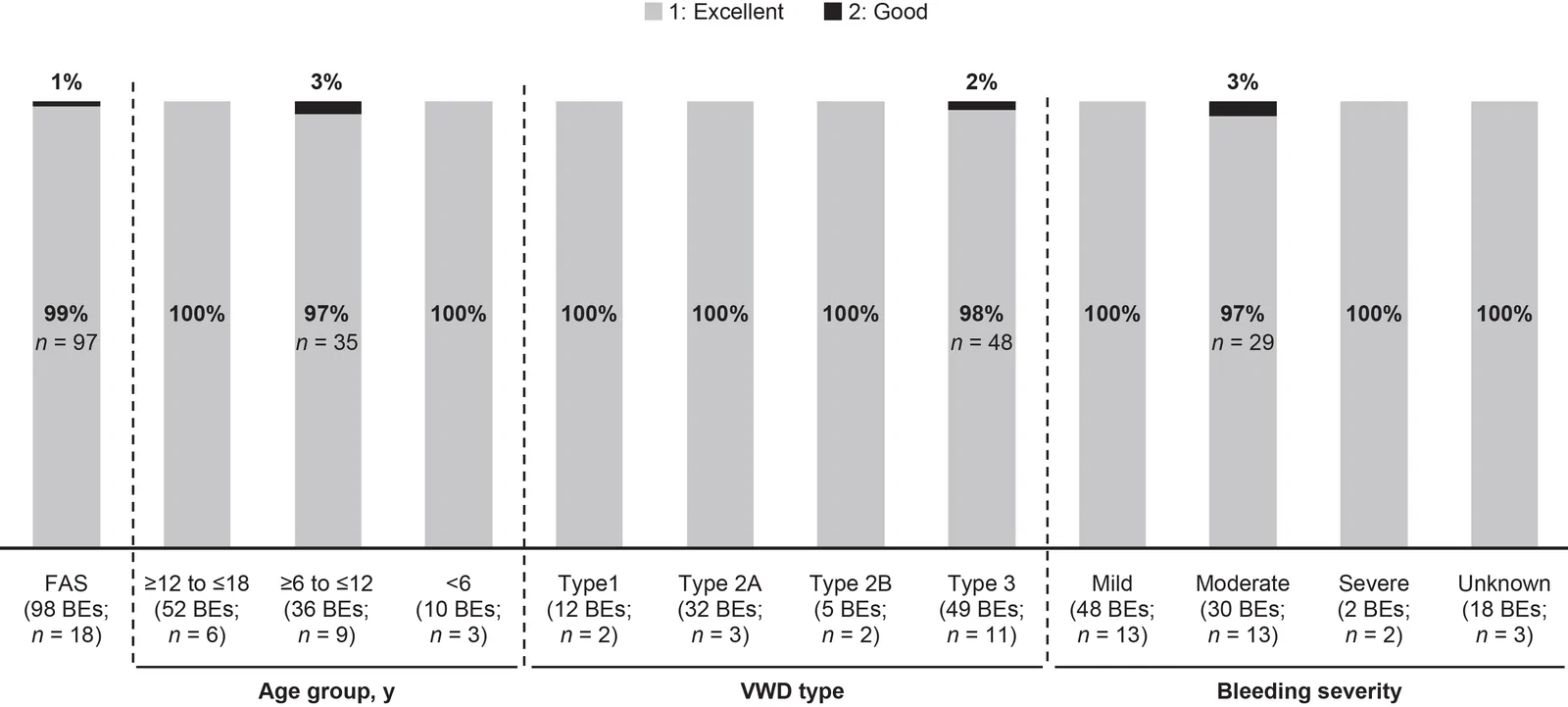
Hemostatic efficacy ratings by BE in the phase 3 study (FAS). Efficacy ratings were available for 98 of 104 treated BEs. Percentages are based on BEs with known hemostatic efficacy ratings. BE, bleeding event; FAS, full analysis set; VWD, von Willebrand disease.

The primary end point of treatment success was achieved in all 18 (100%) patients (95% CI, 81.5-100.0). The mean (SD) efficacy rating score according to a predefined 4-point scale was 1.01 (0.04), and the mean (SD) number of treated BEs per patient was 5.8 (5.0) (Table 3). The secondary end point, based on individual BE assessments, showed treatment hemostatic efficacy for all 98 (100%) BEs with nonmissing ratings; all reported efficacy ratings were assessed as either excellent or good, with excellent being reported for the majority (99%) of BEs. Efficacy results were consistent across age groups, VWD types, bleeding severity categories, and BE locations (Figure 2 and Supplementary Table 4).

### 3.3 Drug consumption

In the phase 3 study, most BEs (82%) resolved with 1 infusion of rVWF, with a similar percentage in each age group (Table 4). The median (range) total dose of rVWF per event was 51.0 (17.6-365.9) IU/kg. This total dose was notably higher in patients aged <6 years driven by 1 outlier. This individual experienced 2 moderate joint BEs in the left ankle (1 traumatic and 1 spontaneous) that were treated with 9 and 8 infusions of rVWF, respectively, including doses to maintain hemostasis after the bleeding stopped. The overall mean (SD) rVWF dose per infusion per event was 48.4 (8.1) IU/kg (median [range]: 48.5 [17.6-63.0] IU/kg) and was similar across age groups. rFVIII was administered for 28 of the 104 BEs (27%) in 7 patients (39%; VWD type 1, *n* = 1; VWD type 3, *n* = 6), all of whom received a single infusion of rFVIII per bleed. The mean (SD) total dose of rFVIII per event was 30.8 (8.6) IU/kg and was similar across age groups (Table 4). Of the 28 BEs for which rFVIII was administered, the most common locations were recorded as joints (9 bleeds) and mucosal nose (8 bleeds) (Supplementary Table 5). Fifteen of the bleeds for which treatment included rFVIII were mild, 12 were moderate, and 1 was of unknown severity (Table 4).

**Table 4:** Consumption of rVWF and rFVIII in the phase 3 study (full analysis set).

Parameter	Full analysis set (*N* = 18)	Age group (y)		
		≥12 to <18 (*n* = 6)	≥6 to <12 (*n* = 9)	<6 (*n* = 3)
Treated nonsurgical bleeding events (*n*)	104	57	37	10
No. of rVWF infusions per bleed, *n* (%)^a^				
1	80 (82)	44 (85)	28 (78)	8 (80)
2	12 (12)	7 (14)	5 (14)	0
3	4 (4)	1 (2)	3 (8)	0
>5	2 (2)	0	0	2 (20)
Missing	6	5	1	0
Total rVWF dose per bleed (IU/kg)	98 bleeds	52 bleeds	36 bleeds	10 bleeds
Mean (SD)	64.4 (48.3)	56.8 (22.6)	62.6 (32.2)	110.5 (124.7)
Median (range)	51.0 (17.6-365.9)	49.8 (17.6-145.5)	51.9 (18.3-169.8)	53.0 (41.2-365.9)
Average rVWF dose per infusion per bleed (IU/kg)	98 bleeds	52 bleeds	36 bleeds	10 bleeds
Mean (SD)	48.4 (8.1)	48.4 (7.5)	48.0 (9.0)	49.4 (8.0)
Median (range)	48.5 (17.6-63.0)	48.5 (17.6-59.7)	51.3 (18.3-63.0)	49.7 (40.7-62.4)
Bleeds treated with rFVIII in addition to rVWF, *n* (%)	28 (27)	15 (26)	9 (24)	4 (40)
Bleeds treated with 1 rFVIII infusion, *n* (%)	28 (100)	15 (100)	9 (100)	4 (100)
Total rFVIII dose per bleed (IU/kg)	28 bleeds	15 bleeds	9 bleeds	4 bleeds
Mean (SD)	30.8 (8.6)	32.2 (8.8)	27.5 (9.7)	32.8 (1.5)
Median (range)	32.9 (9.1-45.0)	35.4 (18.0-42.7)	26.1 (9.1-45.0)	32.9 (30.8-34.5)

	Bleed severity			
	Minor/mild (*n* = 13)	Moderate (*n* = 13)	Major/severe (*n* = 2)	Unknown (*n* = 3)
Treated nonsurgical bleeding events (*n*)	48	31	2	23
No. of rVWF infusions per bleed, *n* (%)^a^				
1	37 (84)	23 (77)	1 (50)	19 (86)
2	6 (14)	4 (13)	0	2 (9)
3	1 (2)	1 (3)	1 (50)	1 (5)
>5	0	2 (7)	0	0
Missing	4	1	0	1
Total rVWF dose per bleed (IU/kg)	44 bleeds	30 bleeds	2 bleeds	22 bleeds
Mean (SD)	55.1 (24.8)	79.4 (75.4)	108.4 (86.8)	58.6 (24.5)
Median (range)	50.6 (17.6-156.7)	53.0 (42.7-365.9)	108.4 (47.0-169.8)	48.5 (46.9-145.5)
Average rVWF dose per infusion per bleed (IU/kg)	44 bleeds	30 bleeds	2 bleeds	22 bleeds
Mean (SD)	46.4 (10.4)	50.0 (6.2)	51.8 (6.8)	49.7 (3.3)
Median (range)	47.1 (17.6-59.7)	51.3 (37.9-62.4)	51.8 (47.0-56.6)	48.5 (46.9-63.0)
Bleeds treated with rFVIII in addition to rVWF, *n* (%)	15 (31)	12 (39)	0	1 (4)
Bleeds treated with 1 rFVIII infusion, *n* (%)	15 (100)	12 (100)	0	1 (100)
Total rFVIII dose per bleed (IU/kg)	15 bleeds	12 bleeds	0 bleeds	1 bleed
Mean (SD)	27.6 (9.4)	34.2 (6.2)	*–*	36.3 (–)
Median (range)	26.1 (9.1-42.7)	34.6 (18.0-45.0)	*–*	36.3 (36.3-36.3)

	VWD type			
	1	2A	2B	3
Treated nonsurgical bleeding events (*n*)	12	37	5	50
No. of rVWF infusions per bleed, *n* (%)^a^				
1	9 (75)	31 (89)	4 (80)	36 (78)
2	2 (17)	3 (9)	0	7 (15)
3	1 (8)	1 (3)	1 (20)	1 (2)
>5	0	0	0	2 (4)
Missing	0	2	0	4
Total rVWF dose per bleed (IU/kg)	12 bleeds	35 bleeds	5 bleeds	46 bleeds
Mean (SD)	66.9 (36.7)	55.1 (22.1)	76.7 (52.2)	69.5 (63.1)
Median (range)	53.1 (18.3-156.7)	48.5 (17.6-145.5)	51.9 (50.8-169.8)	51.6 (25.5-365.9)
Average rVWF dose per infusion per bleed (IU/kg)	12 bleeds	35 bleeds	5 bleeds	46 bleeds
Mean (SD)	49.6 (10.6)	48.2 (6.5)	54.0 (3.5)	47.6 (8.7)
Median (range)	52.2 (18.3-63.0)	48.5 (17.6-57.9)	51.9 (50.8-58.9)	47.7 (25.5-62.4)
Bleeds treated with rFVIII in addition to rVWF, *n* (%)	7 (58)	0	0	21 (42)
Bleeds treated with 1 rFVIII infusion, *n* (%)	7 (100)	0	0	21 (100)
Total rFVIII dose per bleed (IU/kg)	7 bleeds	0 bleeds	0 bleeds	21 bleeds
Mean (SD)	24.7 (8.0)	—	—	32.8 (7.9)
Median (range)	26.1 (9.1-36.3)	—	—	34.5 (18.0-45.0)

rFVIII, recombinant factor VIII; rVWF, recombinant von Willebrand factor; VWD, von Willebrand disease.

a Percentages were based on bleeding events with known number of infusions.

### 3.4 PK and PD analyses

VWF plasma concentrations increased rapidly after a single infusion of 50 IU/kg rVWF. Median levels of VWF:RCo, VWF:Ag, and VWF:CB were highest at 15 minutes after infusion, decreased steadily through 12 hours, then decreased gradually to 48 hours, and remained stable through the last assessment at 96 hours (Supplementary Figure 2). Regarding PD, median levels of FVIII:C showed a trend of increase through the 30-hour assessment, reflecting stabilization of FVIII by rVWF, and then a decrease through the 96-hour assessment.

The PK/PD exposure parameters derived from combined concentration data across time points by age cohort with and without individual baseline correction were evaluated following rVWF infusions to construct an individual PK profile or to collect data on individual PK (Supplementary Table 6). Slightly lower VWF:Ag, VWF:CB, and FVIII:C levels were observed in patients aged <6 years, without causing any discernible difference in the number of infusions or average doses per kilogram administered to these patients. However, no conclusion can be drawn because of the sparse samples used in the calculation.

### 3.5 Safety

Overall, 122 TEAEs were reported in 23 (92%) patients, with 5 (20%) patients reporting 6 serious TEAEs (*Yersinia* sp. infection; obsessive-compulsive disorder; vascular device infection; pyrexia; urinary tract infection; and traumatic hematoma) (Table 5). The majority of the TEAEs were mild, with 2 (8.0%) patients experiencing 3 severe TEAEs. Only 1 TEAE (nausea; moderate severity) was considered by the investigator to be related to rVWF. No serious AEs were considered to be related to study treatment. No thromboembolic events, allergic reaction TEAEs, or severe hypersensitivity reaction TEAEs were reported. There were no deaths or TEAEs leading to treatment discontinuation. No neutralizing antibodies to VWF or FVIII; binding antibodies to VWF; or antibodies to Chinese hamster ovary proteins, murine IgG, or recombinant furin were detected.

**Table 5:** Summary of TEAEs in pediatric patients receiving OD study drug in the phase 3 study (safety analysis set).

Adverse events	Phase 3 study (*N* = 25)		Phase 3b rollover pediatric OD treatment cohort (*N* = 16)	
	Patients, *n* (%)	Events (*n*)	Patients, *n* (%)	Events (*n*)
Any TEAEs	23 (92)	122	15 (94)	117
Treatment-related TEAEs				
TEAEs related to rVWF^a^	1 (4)	1^a^	0	0
TEAEs related to rFVIII	0	0	0	0
TEAEs related to study procedures	0	0	0	0
TEAEs temporally associated with rVWF	9 (36)	13	4 (25)	7
TEAEs temporally associated with rFVIII	3 (12)	3	2 (25)	3
Severity of TEAEs				
Mild	20 (80)	81	14 (88)	87
Moderate	16 (64)	38	11 (69)	29
Severe	2 (8)	3	1 (6)	1
Serious TEAEs	5 (20)	6^b^	3 (19)	5^c^
Treatment-related serious TEAEs	0	0	0	0
TEAEs leading to treatment discontinuation	0	0	0	0
Deaths	0	0	0	0
TEAEs of interest				
Thromboembolic events	0	0	0	0
Allergic reactions to rVWF or rFVIII	0	0	0	0
Severe hypersensitivity reactions	0^d^	0	0	0
TEAEs in ≥10% of patients in either the phase 3 or the phase 3b study				
Pyrexia	4 (16)	9	2 (13)	2
Upper respiratory tract infection	4 (16)	6	6 (38)	9
Vomiting	4 (16)	5	4 (25)	6
Cough	3 (12)	4	4 (25)	7
Diarrhea	3 (12)	6	1 (6)	1
Iron deficiency anemia	3 (12)	3	2 (13)	2
Rash	3 (12)	3	0	0
Oropharyngeal pain	2 (8)	2	3 (19)	3
Headache	1 (4)	1	2 (13)	4
Iron deficiency	1 (4)	1	2 (13)	2
Nasal congestion	1 (4)	1	2 (13)	2
Pain in extremity	1 (4)	1	2 (13)	2
COVID-19	0	0	8 (50)	8
Coronavirus infection	0	0	3 (19)	3
Alanine aminotransferase increased	0	0	2 (13)	2
Limb injury	0	0	2 (13)	2
Sunburn	0	0	2 (13)	2
Viral infection	0	0	2 (13)	2

OD, on-demand; rFVIII, recombinant factor VIII; rVWF, recombinant von Willebrand factor; TEAE, treatment-emergent adverse event.

a Nausea of moderate severity in 1 patient.

b Two serious TEAEs were mild (*Yersinia* sp. infection and obsessive-compulsive disorder), 1 was moderate (vascular device infection), and 3 were severe (pyrexia, urinary tract infection, and traumatic hematoma).

c One patient with a fall and medical device site extravasation; 1 patient with coronavirus infection and hypotension; and 1 patient with spinal compression fracture.

d One patient experienced mild nonserious pruritus, which was identified as a potential hypersensitivity reaction per broad standardized Medical Dictionary for Regulatory Activities query criteria. The event (skin sensitivity to mediport dressing) resolved within 1 day and was not considered to be related to study treatment.

### 3.6 Phase 3b study rollover on-demand treatment cohort

In the phase 3b rollover pediatric on-demand treatment cohort of the continuation study, 16 of 19 rollover patients received study drug for on-demand treatment during the study and were included in the SAS and FAS (Figure 1). Baseline characteristics for patients in the rollover cohort are shown in Table 1 and Supplementary Table 3. In the 16 treated patients, a hemostatic efficacy rating of excellent or good was achieved for 100% of 166 treated BEs with nonmissing ratings, and efficacy was consistent across all 3 age groups. BEs were treated with a mean (SD) of 1.1 (0.7) infusions of rVWF per bleed (without or with rFVIII) (Supplementary Table 7). rFVIII was administered in addition to rVWF for 46 of 167 treated BEs (28%) in 7 patients. All cases in the continuation study used 1 or 2 rFVIII infusions per bleed (mean [SD]: 1.1 [0.25]).

Among the 16 treated rollover patients, 15 (94%) patients reported 117 TEAEs, with 3 (19%) patients reporting 5 serious TEAEs (fall, medical device site extravasation, coronavirus infection, hypotension, and spinal compression fracture) (Table 5). None of the reported TEAEs were considered to be related to study treatment. No thromboembolic events, allergic reaction TEAEs, or severe hypersensitivity reaction TEAEs were reported. No neutralizing antibodies to VWF or FVIII; binding antibodies to VWF; or antibodies to Chinese hamster ovary proteins, murine IgG, or recombinant furin were detected. One patient in the continuation study tested positive for binding IgG antibody to FVIII at month 18 before the first rVWF treatment in this study and then after treatment at the month 24, month 33, and end of study visits. The patient did not receive rFVIII during the study and the FVIII-binding IgG antibody did not have a notable impact on the efficacy and safety responses in this patient. rVWF remained efficacious for this patient: a hemostatic efficacy rating of excellent was achieved for the treatment of BEs that the patient experienced (2 BEs; both moderate severity).

## 4 Discussion

In the final analysis of data from the on-demand arm of the phase 3 study, the primary end point of treatment success was met in all 18 (100%) pediatric patients who experienced a BE treated with rVWF (without or with rFVIII), during ∼12 to 17 months of the on-demand treatment period. A hemostatic efficacy rating of excellent or good was achieved in 100% of assessed treated BEs, with excellent being reported for the majority (99.0%) of BEs. Consistent efficacy was demonstrated by pediatric age group, VWD type, bleeding severity, and bleeding location. Most BEs (82%) resolved with 1 infusion of rVWF, and most (73%) did not require an rFVIII infusion in addition to rVWF for bleeding treatment. The mean (SD) rVWF dose per infusion per bleed was 48.4 (8.1) IU/kg, consistent with the recommended initial dose for adult on-demand treatment (40-80 IU/kg) [19,20]. In the phase 3b continuation study, rVWF continued to show similar efficacy for on-demand treatment in 16 patients for an additional treatment period of up to 3 years, with all rated BEs having a hemostatic efficacy rating of excellent or good. BEs resolved with a mean 1.1 rVWF infusions, and the majority of these bleeds did not require rFVIII.

The results for children and adolescents in our study align with those previously reported in adults receiving on-demand therapy. For example, in a pivotal phase 3 study in 22 patients with severe VWD, on-demand treatment with 1 infusion of rVWF (and with rFVIII in 94% of these treatments) was effective in 82% of bleeds, with treatment success achieved in 100% of patients [25]. The present studies also demonstrate hemostatic efficacy with rVWF without the use of rFVIII in pediatric patients, consistent with findings in adults [25,31] and in agreement with its role in stabilizing endogenous FVIII. This is also aligned with the current dosing recommendation that rVWF may be given without rFVIII when an immediate rise in FVIII is not required or when baseline FVIII levels are adequate to achieve hemostasis [19,20]. In the phase 3 study, 7 patients received rFVIII along with rVWF. Patients were treated according to the protocol dosing guidance, so would have received rFVIII at the investigator’s discretion to maintain hemostasis due to low baseline FVIII levels, as 6 of the 7 patients had type 3 VWD with low baseline FVIII. It is also possible that some investigators may have dosed rFVIII based on their clinical practice. The observation that many cases of rFVIII coadministration in our phase 3 study were for BEs of mild severity indicates that a cautious approach was being taken. Overall, data support the flexibility of dosing FVIII while treating BEs with rVWF. In contrast to the varying concentrations of VWF/FVIII ratios in plasma-derived VWF products, rVWF contains no FVIII, thus allowing the physician to optimize the therapy without or with rFVIII for each patient. This reduces the risk of excessive FVIII plasma levels and the associated risk of thrombosis, especially when used for the repeated dosing that is required to treat major and difficult-to-manage BEs.

The PK/PD profile of on-demand rVWF showed a response pattern similar to the known rVWF PK/PD profile in adults [25,29]. The levels of FVIII:C in our study peaked at a later time than VWF activity, which is expected based on the disposition and PK/PD relationship of rVWF. However, due to the limitations of a small sample size and a sparse sampling approach, additional population PK analyses are warranted to characterize the PK/PD of rVWF in the pediatric population.

Overall, no new safety concerns were identified during up to 4 years of rVWF treatment, compared with reports of rVWF safety in adults [25,29,31,32]. The only TEAE considered to be related to rVWF in the present studies was a case of moderate nausea in the phase 3 study. Nausea is an existing serious adverse event of rVWF identified from the adult trials.

The studies recruited a sample of pediatric patients that was well distributed across 3 types of VWD and 3 age groups, including 5 young children aged <6 years, among whom 2 were infants aged <2 years, adding a considerable amount of safety and efficacy data in support of the evaluation of the rVWF benefit–risk profile in pediatric patients with VWD. In addition, the continuation study allowed the long-term safety and efficacy of rVWF to be assessed for up to a total of 4 years across 2 studies. Limitations of this study included the open-label, uncontrolled design, and the limited patient sample, although it is justified and acceptable according to regulatory guidelines for this rare disease. In addition, investigator-rated hemostatic efficacy is inherently subjective and may be influenced by expectation bias. However, hemostatic efficacy often requires real-time clinical assessment and was evaluated using an accepted approach common to bleeding disorder studies.

In conclusion, rVWF without and with rFVIII was demonstrated to be efficacious for the treatment of nonsurgical BEs in pediatric patients with VWD, with consistent efficacy across all pediatric age groups, VWD types, bleeding severities, and bleeding locations. Most BEs resolved after 1 infusion of rVWF, and the majority of bleeds did not require additional rFVIII. It remains of interest to model FVIII levels without and with additional FVIII treatment. Across the phase 3 and phase 3b studies, efficacy was maintained for up to 4 years of the on-demand rVWF treatment period. rVWF was well tolerated, and no new adverse drug reactions were identified in the pediatric population of the studies. Although the single-arm, open-label design of this trial precludes benchmarking of outcomes against plasma-derived VWF products, the findings support the use of rVWF as an efficacious treatment option, with no new safety concerns, for nonsurgical BEs in pediatric patients with VWD.
