# Supplementary material for: Efficacy and safety of recombinant von Willebrand factor in on-demand treatment of children with von Willebrand disease: up to 4 years of phase 3/3b follow-up
Source: Res Pract Thromb Haemost. 2026 Jul 13;10(5):106817. doi: 10.1016/j.rpth.2026.106817 (PMC13499520; doi:10.1016/j.rpth.2026.106817)
Supplement: Supplementary file 1 [file mmc1.docx]

Supplementary Information

**Efficacy and safety of recombinant von Willebrand factor in on-demand treatment of children with von Willebrand disease: up to 4 years of phase 3/3b follow-up**

**Authors:** Shayla Bergmann^1^ | Sanjay Ahuja^2^ | Canan Albayrak^3^ | Marjon H. Cnossen^4^ | Amy L. Dunn^5^ | Veerle Labarque^6^ | Matteo Luciani^7^ | Christoph Male^8^ | Eric S. Mullins^9^ | Sophie Susen^10^ | Pascual Marco Vera^11^ | Ali G. Mokdad^12^ | Yi Wang^12^ | Josh Weng^12^ | Jingmei Zhang^12^

**Affiliations:**

^1^Department of Pediatrics, Medical University of South Carolina, Charleston, South Carolina, USA

^2^Innovative Hematology, Inc., Indiana Hemophilia and Thrombosis Center, Indianapolis, Indiana, USA

^3^Division of Pediatric Hematology and Oncology, Ondokuz Mayis University Faculty of Medicine, Samsun, Turkey

^4^Department of Pediatric Hematology and Oncology, Erasmus Medical Center Sophia Children's Hospital, University Medical Center Rotterdam, Rotterdam, the Netherlands

^5^Department of Hematology, Oncology, Bone Marrow Transplant, Nationwide Children's Hospital and The Ohio State University College of Medicine, Columbus, Ohio, USA

^6^Department of Pediatric Hemato-Oncology, University Hospitals Leuven; Centre of Molecular and Vascular Biology, Katholieke Universiteit Leuven, Leuven, Belgium

^7^Onco-Hematology, Cell and Gene Therapy and Bone Marrow Transplant Clinic Area, Bambino Gesù Children's Hospital, Istituto di Ricovero e Cura a Carattere Scientifico, Rome, Italy

^8^Department of Paediatrics, Medical University of Vienna, Vienna, Austria

^9^Division of Hematology, Cincinnati Children's Hospital Medical Center; University of Cincinnati College of Medicine, Cincinnati, Ohio, USA

^10^University of Lille, Inserm, Centre Hospitalier Universitaire Lille, Institut Pasteur de Lille, Lille, France

^11^Biomedical Research Institute (Instituto de Investigación Sanitaria y Biomédica de Alicante); Clinical Medicine Department, Miguel Hernández University, Alicante, Spain

^12^Takeda Development Center Americas, Inc, Cambridge, Massachusetts, USA

**Correspondence**

Email: bergmans@musc.edu

**Journal:** *Research and Practice in Thrombosis and Haemostasis*

**This file includes:**

Supplementary Figures S1–2

Supplementary Tables S1–7

# Supplementary material

## Supplementary Figures

**SUPPLEMENTARY FIGURE S1** Study design. ^a^ Occurred after surgery in emergency surgery arm. ^b^ Initial dose of 40-60 IU/kg rVWF and up to 80 IU/kg for major bleeding events. EOS, end of study; OD, on demand; PK, pharmacokinetic; rFVIII, recombinant factor VIII; rVWF, recombinant von Willebrand factor.

**SUPPLEMENTARY FIGURE S2** Observed pharmacokinetic/pharmacodynamic profiles from patients across age groups in the phase 3 study. Patients had an initial pharmacokinetic assessment after a washout of ≥168 hours before infusion of 50 ± 5 IU/kg rVWF. Median levels are derived from the combination of all patients across age groups. FVIII:C, factor VIII coagulation activity; Pre-inf, preinfusion; rVWF, recombinant von Willebrand factor; VWF:Ag, von Willebrand factor antigen; VWF:CB, von Willebrand factor collagen binding capacity; VWF:RCo, von Willebrand factor ristocetin cofactor.

## Supplementary Tables

**SUPPLEMENTARY TABLE S1** Inclusion and exclusion criteria for the phase 3 study.

| **Inclusion criteria** | **Exclusion criteria** |
| --- | --- |
| 1. Diagnosis of severe VWD (defined as VWF:RCo <20%):    a. Type 1 (VWF:RCo <20 IU/dL); or    b. Type 2A (VWF:RCo <20 IU/dL), type 2B (as diagnosed by genotype), type 2N (FVIII:C <10% and historically documented genetics), type 2M; or    c. Type 3 (VWF:Ag ≤3 IU/dL) 2. Age 0 to <18 years at the time of screening 3. The patient had provided assent (if appropriate) and legally authorized representative(s) had provided informed consent 4. If female of childbearing potential, patients presented with a negative serum pregnancy test 5. If applicable, the patient agreed to employ adequate birth control measures for the duration of the study 6. The patient and/or legal representative were willing and able to comply with the requirements of the protocol, which was also confirmed based on a prescreening evaluation held between the investigator and the sponsor, to ensure no imminent risk was present that could challenge the patient’s compliance with the study requirements   Additional inclusion criteria for both previously treated patients and patients undergoing surgery:   1. Unable to tolerate or inadequately responsive to DDAVP 2. Patient had ≥1 documented bleed requiring VWF coagulation factor replacement therapy during the 12 months before enrollment, and overall, ≥3 exposure days historically to VWF replacement therapy   Additional inclusion criterion for previously untreated patients:   1. Patient had not received prior VWF coagulation factor replacement therapy | 1. Diagnosis of pseudo-VWD or another hereditary or acquired coagulation disorder (eg, qualitative and quantitative platelet disorders or elevated prothrombin time/INR >1.4) 2. History or presence of a VWF inhibitor at screening 3. History or presence of a FVIII inhibitor with a titer ≥0.4 BU (Nijmegen assay) or ≥0.6 BU (Bethesda assay) 4. Documented history of a VWF:RCo half-life <6 hours 5. Known hypersensitivity to any of the components of the study drug, such as mouse or hamster proteins 6. Medical history of immunological disorders, excluding seasonal allergic rhinitis/conjunctivitis/asthma, food allergies, or animal allergies 7. Medical history of a thromboembolic event 8. Human immunodeficiency virus positive, with an absolute CD4 count <200/mm^3^ 9. In the judgment of the investigator, the patient had another clinically significant concomitant disease (eg, uncontrolled hypertension, cancer) that may pose additional risk for the patient 10. Diagnosis of significant liver disease, as evidenced by, but not limited to, any of the following: serum alanine aminotransferase 5× the upper limit of normal, hypoalbuminemia, portal vein hypertension (eg, presence of otherwise unexplained splenomegaly, history of esophageal varices), or liver cirrhosis classified as Child-Pugh B or C 11. Diagnosis of renal disease, with a serum creatinine level ≥2.5 mg/dL 12. Immunomodulatory drug treatment other than anti-retroviral chemotherapy (eg, α‑interferon or corticosteroid agents) at a dose equivalent to hydrocortisone >10 mg/day (excluding topical treatment such as ointments and nasal sprays), within 30 days before signing informed consent (or assent, if appropriate) 13. If female, the patient was pregnant or lactating at the time informed consent (or assent, if appropriate) was obtained 14. The patient had participated in another clinical study involving a study drug, other than rVWF without or with rFVIII, or an investigational device within 30 days before enrollment, or was scheduled to participate in another clinical study involving a study drug other than rVWF or investigational device during the course of this study 15. Patient’s legal representative was a family member or employee of the investigator |

BU, Bethesda units; DDAVP, desmopressin; FVIII, factor VIII; FVIII:C, factor VIII coagulation activity; INR, international normalized ratio; rFVIII, recombinant factor VIII; rVWF, recombinant von Willebrand factor; VWD, von Willebrand disease; VWF, von Willebrand factor; VWF:Ag, von Willebrand factor antigen; VWF:RCo, von Willebrand factor ristocetin cofactor.

**SUPPLEMENTARY TABLE S2** Efficacy rating criteria.

| **Rating** | **Minor/mild and moderate bleeding events** | **Major/severe bleeding events** |
| --- | --- | --- |
| Excellent (=1) | - Actual number of infusions ≤ estimated number of infusions required to treat that bleeding event - No additional VWF-containing coagulation factor–containing product required | - Actual number of infusions ≤ estimated number of infusions required to treat that bleeding event - No additional VWF-containing coagulation factor–containing product required |
| Good (=2) | - 1-2 infusions greater than estimated required to control that bleeding event - No additional VWF-containing coagulation factor–containing product required | - <1.5× infusions greater than estimated required to control that bleeding event - No additional VWF-containing coagulation factor–containing product required |
| Moderate (=3) | - 3 or more infusions greater than estimated used to control that bleeding event - No additional VWF-containing coagulation factor–containing product required | - ≥1.5× more infusions greater than estimated used to control that bleeding event - No additional VWF-containing coagulation factor–containing product required |
| None (=4) | - Severe uncontrolled bleeding or intensity of bleeding not changed - Additional VWF-containing coagulation factor–containing product required | - Severe uncontrolled bleeding or intensity of bleeding not changed - Additional VWF-containing coagulation factor–containing product required |

VWF, von Willebrand factor.

Examples of minor/mild bleeding events include epistaxis, oral bleeding, and menorrhagia. Examples of major/severe bleeding events include severe or refractory epistaxis, menorrhagia, gastrointestinal bleeding, central nervous system trauma, hemarthrosis, and traumatic hemorrhage.

**SUPPLEMENTARY TABLE S3** Baseline characteristics (full analysis set).

|  | **Phase 3 study**  **(*N* = 18)** | **Phase 3b rollover pediatric OD treatment cohort**  **(*N* = 16)** |
| --- | --- | --- |
| **Age, y** |  |  |
| Mean (SD) | 10.4 (5.0) | 10.4 (5.4) |
| Median (range) | 11.0 (1-17) | 10.5 (2-18) |
| **Age category, *n* (%)** |  |  |
| 18 y | 0 | 2 (13) |
| ≥12 to <18 y | 6 (33) | 6 (38) |
| ≥6 to <12 y | 9 (50) | 5 (31) |
| <6 y | 3 (17) | 3 (19) |
| **Sex, *n* (%)** |  |  |
| Female | 11 (61) | 9 (56) |
| Male | 7 (39) | 7 (44) |
| **Patient of childbearing potential, *n* (%)^a^** |  |  |
| Yes | 4 (36) | 3 (33) |
| **VWD type, *n* (%)** |  |  |
| 1 | 2 (11) | 4 (25) |
| 2A | 3 (17) | 1 (6) |
| 2B | 2 (11) | 3 (19) |
| 3 | 11 (61) | 8 (50) |
| **Age category by VWD type (*n*)** |  |  |
| ≥12 to <18 y | Types: 2A/B (2), 3 (4) | Types: 1 (1), 2A/B (3), 3 (4) |
| ≥6 to <12 y | Types: 1 (1), 2A/B (3), 3 (5) | Types: 2B (1), 3 (4) |
| <6 y | Types: 1 (1), 3 (2) | Type: 1 (3) |
| **Body mass index, kg/m^2^** |  |  |
| Mean (SD) | 20.7 (7.1) | 21.7 (7.8) |
| Median (range) | 18.4 (13.4-40.7) | 19.9 (13.4-40.6) |
| **Race, *n* (%)^b^** |  |  |
| White | 15 (94) | 14 (93) |
| Multiple | 1 (6) | 1 (7) |
| Not reported | 2 | 1 |
| **Ethnicity, *n* (%)^b^** |  |  |
| Hispanic or Latino | 1 (6) | 1 (7) |
| Not Hispanic or Latino | 16 (94) | 14 (93) |
| Not reported | 1 | 1 |

OD, on demand; VWD, von Willebrand disease.

^a^ Percentages based on number of female patients.

^b^ Percentages calculated with the missing data numbers removed.

**SUPPLEMENTARY TABLE S4** Efficacy by bleeding event location in the phase 3 study (full analysis set) [1].

| **Bleeding events by location** | **Median (range) no. of infusions of rVWF** | **Median (range) no. of infusions of rFVIII** | **Rating (%)** |
| --- | --- | --- | --- |
| **Joint**  (*n* = 17) | 1 (1-9) | 1 (1-1) | Excellent (100%)  Good (0%) |
| **Mucosal: nasopharyngeal**  (*n* = 25) | 1 (1-3) | 1 (1-1) | Excellent (100%)  Good (0%) |
| **Mucosal: mouth and oral cavity** (*n* = 14) | 1 (1-3) | N/A | Excellent (93%)  Good (7%) |
| **Mucosal: genital tract female** (*n* = 12) | 1 (1-1) | 1 (1-1) | Excellent (100%)  Good (0%) |
| **Muscle/skin/soft tissue**  (*n* = 14) | 1 (1-3) | 1 (1-1) | Excellent (100%)  Good (0%) |
| **Multiple/other**  (*n* = 21) | 1 (1-2) | 1 (1-1) | Excellent (100%)  Good (0%) |

N/A, not applicable; rFVIII, recombinant factor VIII; rVWF, recombinant von Willebrand factor.

[1] Takeda Pharmaceuticals USA, Inc. Vonvendi. Prescribing information, https://www.shirecontent.com/PI/PDFs/VONVENDI_USA_ENG.pdf; 2025 [accessed 30 October 2025].

**SUPPLEMENTARY TABLE S5** Drug consumption by bleeding location in the phase 3 study (full analysis set).

|  | **Mucosal nose** | **Mucosal gum** | **Mucosal mouth** | **Skin** | **Muscle** | **Soft tissue** | **Joint** | **Multiple/other^a^** | **Unknown** |
| --- | --- | --- | --- | --- | --- | --- | --- | --- | --- |
| **Patients with bleeds, *n*** | 7 | 5 | 2 | 3 | 4 | 3 | 6 | 13 | 1 |
| **Treated bleeds, *n*** | 25 | 11 | 3 | 4 | 7 | 3 | 17 | 33 | 1 |
| **No. of rVWF infusions per bleed, *n* (%)^b^** |  |  |  |  |  |  |  |  |  |
| 1 | 21 (88) | 8 (73) | 2 (100) | 4 (100) | 6 (86) | 2 (67) | 10 (63) | 26 (87) | 1 (100) |
| 2 | 2 (8) | 2 (18) | 0 | 0 | 1 (14) | 0 | 3 (19) | 4 (13) | 0 |
| 3 | 1 (4) | 1 (9) | 0 | 0 | 0 | 1 (33) | 1 (6) | 0 | 0 |
| >5 | 0 | 0 | 0 | 0 | 0 | 0 | 2 (13) | 0 | 0 |
| Missing | 1 | 0 | 1 | 0 | 0 | 0 | 1 | 3 | 0 |
| **Total rVWF dose per bleed, IU/kg** | *[24 bleeds]* | *[11 bleeds]* | *[2 bleeds]* | *[4 bleeds]* | *[7 bleeds]* | *[3 bleeds]* | *[16 bleeds]* | *[30 bleeds]* | *[1 bleed]* |
| Mean (SD) | 57.4 (27.4) | 58.9 (25.0) | 47.1 (15.4) | 49.9 (3.0) | 47.4 (28.5) | 90.7 (69.0) | 102.3 (99.8) | 56.6 (15.7) | 52.0 (–) |
| Median (range) | 49.6 (18.3-156.7) | 51.3 (37.9-113.7) | 47.1 (36.2-57.9) | 49.9 (46.9-53.0) | 50.8 (17.6-101.6) | 59.7 (42.7-169.8) | 56.4 (44.3-365.9) | 52.1 (37.9-102.5) | 52.0 (52.0-52.0) |
| **Average rVWF dose per infusion per bleed, IU/kg** | *[24 bleeds]* | *[11 bleeds]* | *[2 bleeds]* | *[4 bleeds]* | *[7 bleeds]* | *[3 bleeds]* | *[16 bleeds]* | *[30 bleeds]* | *[1 bleed]* |
| Mean (SD) | 48.7 (8.0) | 44.3 (6.4) | 47.1 (15.4) | 49.9 (3.0) | 40.1 (16.2) | 53.0 (9.0) | 48.9 (6.4) | 50.5 (5.8) | 52.0 (–) |
| Median (range) | 49.6 (18.3-62.4) | 43.0 (37.9-51.9) | 47.1 (36.2-57.9) | 49.9 (46.9-53.0) | 50.8 (17.6-58.1) | 56.6 (42.7-59.7) | 48.0 (40.4-59.5) | 49.8 (37.7-63.0) | 52.0 (52.0-52.0) |
| **Bleeds treated with rFVIII in addition to rVWF, *n* (%)** | 8 (33) | 0 | 0 | 0 | 2 (29) | 1 (33) | 9 (56) | 8 (27) | 0 |
| **No. of rFVIII infusions per bleed, *n* (%)^b^** |  |  |  |  |  |  |  |  |  |
| 1 | 8 (100) | 0 | 0 | 0 | 2 (100) | 1 (100) | 9 (100) | 8 (100) | 0 |
| **Total rFVIII dose per bleed, IU/kg** | *[8 bleeds]* | – | – | – | *[2 bleeds]* | *[1 bleed]* | *[9 bleeds]* | *[8 bleeds]* | – |
| Mean (SD) | 26.2 (7.7) | – | – | – | 19.8 (0.8) | 40.8 (–) | 32.4 (7.7) | 35.0 (7.7) | – |
| Median (range) | 26.6 (9.1-34.5) | – | – | – | 19.8 (19.3-20.4) | 40.8 (40.8-40.8) | 32.9 (18.0-42.7) | 35.9 (18.0-45.0) | – |

rFVIII, recombinant factor VIII; rVWF, recombinant von Willebrand factor.

^a^ “Other” included bleeds labeled as backside, eye, finger, gastrocnemii, menstrual bleeding, subcutaneous, tibia, tonsil bleeding, tooth extraction, and trauma due to car accident.

^b^ Percentages based on bleeding events with known number of infusions.

**SUPPLEMENTARY TABLE S6** Median PK/PD parameters calculated using noncompartmental analysis in the phase 3 study (PK analysis set).

| **Parameter** | **PK/PD analysis set**  **(*N* = 22)** | **Age group, y** | | | | |
| --- | --- | --- | --- | --- | --- | --- |
|  |  | **≥12 to <18 (*n* = 7)** | **≥6 to <12 (*n* = 10)** | | **<6 (*n* = 5)** | |
| **VWF:RCo** |  |  |  | |  | |
| Without baseline correction |  |  |  | |  | |
| AUC_0-96h_, h·IU/dL | 1708 | 920 | 2029 | | 2064 | |
| C_max_, IU/dL | 75.4 | 78.5 | 106.6 | | 60.5 | |
| T_max_, h | 1 | 1 | 0.25 | | 1 | |
| With baseline correction |  |  |  | |  | |
| AUC_0-96h_, h·IU/dL | 952 | 905 | 1211 | | 857 | |
| C_max_, IU/dL | 62.3 | 78.5 | 106.6 | | 60.5 | |
| T_max_, h | 1 | 1 | 0.25 | | 1 | |
| **VWF:Ag** |  |  |  | |  | |
| Without baseline correction |  |  |  | |  | |
| AUC_0-96h_, h·IU/dL | 2971 | 2857 | 3897 | | 2453 | |
| C_max_, IU/dL | 84 | 105 | 106 | | 78 | |
| T_max_, h | 0.25 | 1 | 0.25 | | 1 | |
| With baseline correction |  |  |  | |  | |
| AUC_0-96h_, h·IU/dL | 1975 | 2267 | 2046 | | 1753 | |
| C_max_, IU/dL | 84 | 91 | 93 | | 78 | |
| T_max_, h | 0.25 | 1 | 0.25 | | 1 | |
| **VWF:CB** |  |  |  | |  | |
| Without baseline correction |  |  |  | |  | |
| AUC_0-96h_, h·IU/dL | 3351 | 3060 | 4122 | | 2744 | |
| C_max_, IU/dL | 117.2 | 153.2 | 166.4 | | 96.2 | |
| T_max_, h | 0.25 | 1 | 0.25 | | 6 | |
| With baseline correction |  |  |  | |  | |
| AUC_0-96h_, h·IU/dL | 2409 | 2775 | 2771 | | 1944 | |
| C_max_, IU/dL | 117.2 | 149.1 | 166.4 | | 90.3 | |
| T_max_, h | 0.25 | 1 | 0.25 | | 1 | |
| **FVIII:C** |  |  |  | |  | |
| Without baseline correction |  |  |  | |  | |
| AUC_0-96h_, h·IU/dL | 4810 | 4712 | 6255 | | 3540 | |
| C_max_, IU/dL | 81 | 91.5 | 94.5 | | 99 | |
| T_max_, h | 30 | 30 | | 30 | | 0.25 |
| With baseline correction |  |  | |  | |  |
| AUC_0-96h_, h·IU/dL | 3216 | 3419 | | 3426 | | 1766 |
| C_max_, IU/dL | 66 | 86.5 | | 75 | | 87 |
| T_max_, h | 30 | 30 | | 12 | | 0.25 |

AUC_0-96h_, area under the plasma concentration/time curve from 0 to 96 hours post infusion; C_max_, maximal plasma concentration; FVIII:C, factor VIII coagulation activity; IU, international units; PD, pharmacodynamic; PK, pharmacokinetic; T_max_, time to maximal plasma concentration; VWF:Ag, von Willebrand factor antigen; VWF:CB, von Willebrand factor collagen binding capacity; VWF:RCo, von Willebrand factor ristocetin cofactor.

Following baseline correction, any preinfusion value was set to 0.

**SUPPLEMENTARY TABLE S7** Drug consumption in the phase 3b continuation rollover on demand treatment cohort (full analysis set).

|  | **Full analysis set**  **(*N* = 16)** | **Age group, y** | | | |
| --- | --- | --- | --- | --- | --- |
|  |  | **18**  **(*n* = 2)** | **≥12 to ≤18 (*n* = 6)** | **≥6 to <12 (*n* = 5)** | **<6 (*n* = 3)** |
| **Treated nonsurgical bleeds, *n*** | 167 | 29 | 55 | 48 | 35 |
| **No. of rVWF infusions per bleed** |  |  |  |  |  |
| Mean (SD) | 1.1 (0.7) | 1.2 (0.8) | 1.0 (0.2) | 1.2 (1.0) | 1.0 (0.2) |
| Median (range) | 1.0 (1-8) | 1.0 (1-5) | 1.0 (1-2) | 1.0 (1-8) | 1.0 (1-2) |
| **Total rVWF dose per bleed, IU/kg** | *[165 bleeds]* | *[29 bleeds]* | *[55 bleeds]* | *[48 bleeds]* | *[33 bleeds]* |
| Mean (SD) | 56.1 (33.1) | 59.1 (38.7) | 51.5 (10.0) | 59.4 (51.3) | 56.3 (15.0) |
| Median (range) | 49.8 (28.7-391.7) | 49.4 (36.3-250.3) | 51.0 (28.7-98.1) | 47.6 (38.7-391.7) | 53.3 (46.2-98.6) |
| **Bleeds treated with rFVIII in addition to rVWF, *n* (%)** | 46 (28) | 5 (17) | 37 (67) | 3 (6) | 1 (3) |
| **No. of rFVIII infusions per bleed** |  |  |  |  |  |
| Mean (SD) | 1.1 (0.25) | 1.2 (0.45) | 1.0 (0) | 1.7 (0.58) | 1.0 (-) |
| Median (range) | 1.0 (1-2) | 1.0 (1-2) | 1.0 (1-1) | 2.0 (1-2) | 1.0 (1-1) |
| **Total rFVIII dose per bleed, IU/kg** | *[46 bleeds]* | *[5 bleeds]* | *[37 bleeds]* | *[3 bleeds]* | *[1 bleed]* |
| Mean (SD) | 39.2 (11.1) | 42.7 (15.5) | 36.4 (3.7) | 69.6 (21.3) | 35.9 |
| Median (range) | 37.0 (20.6-90.7) | 35.8 (34.5-70.3) | 37.0 (20.6-39.2) | 70.2 (48.0-90.7) | 35.9 |

rFVIII, recombinant factor VIII; rVWF, recombinant von Willebrand factor.
